# Electronic Nose Based on Independent Component Analysis Combined with Partial Least Squares and Artificial Neural Networks for Wine Prediction

**DOI:** 10.3390/s120608055

**Published:** 2012-06-11

**Authors:** Teodoro Aguilera, Jesús Lozano, José A. Paredes, Fernando J. Álvarez, José I. Suárez

**Affiliations:** 1 Sensory Systems Research Group, University of Extremadura, 06006 Badajoz, Spain; E-Mails: jesuslozano@unex.es (J.L.); japaredesm@unex.es (J.A.P.); fafranco@unex.es (F.J.A.); 2 Industrial Applications of Artificial Intelligence Research Group, 06006 Badajoz, Spain; E-Mail: jmarcelo@unex.es

**Keywords:** independent component analysis, partial least squares, artificial neural networks, electronic nose, wine classification

## Abstract

The aim of this work is to propose an alternative way for wine classification and prediction based on an electronic nose (e-nose) combined with Independent Component Analysis (ICA) as a dimensionality reduction technique, Partial Least Squares (PLS) to predict sensorial descriptors and Artificial Neural Networks (ANNs) for classification purpose. A total of 26 wines from different regions, varieties and elaboration processes have been analyzed with an e-nose and tasted by a sensory panel. Successful results have been obtained in most cases for prediction and classification.

## 1. Introduction

**Figure 1.**
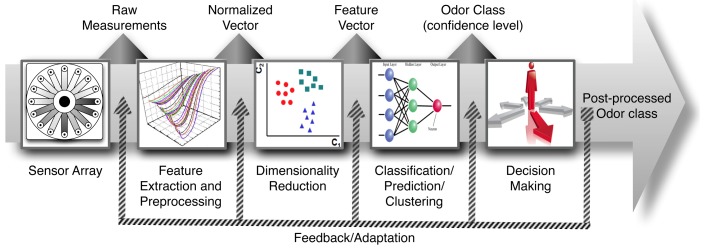
Stages of typical signal processing in an electronic nose.

The most important sensory characteristic in the study of the quality of wine is its aroma. Wine aroma has a great chemical complexity because it is produced by the simultaneous perception of many volatile compounds. This property contributes a 70% of weight in sensory panels with respect to texture and taste. Electronic nose (e-nose) technology is shown as a possibility for aroma profile analysis. Essentially the e-nose is an array of gas sensors with partially overlapped selectivities, a signal collecting unit and a pattern recognition software [1]. On the other hand, pattern analysis constitutes a really important building block in the development of gas sensor instruments given its ability of detecting, identifying and measuring volatile compounds. For these reasons this technology has been proposed as an artificial substitute of the human olfactory system. To successfully design a pattern analysis system for machine olfaction, a rigorous study is necessary on the diverse issues involved in processing multivariate data (see Figure 1): signal preprocessing and feature extraction, dimensionality reduction, classification, regression or clustering and validation [1,2]. A variety of Pattern Recognition and Machine Learning techniques is available for each module and only the preprocessing module is sensor dependent.

The first block in Figure 1 represents the e-nose hardware. After sensor signals have been acquired and saved into the computer, the first computational stage (*i.e.*, signal preprocessing) starts and serves various purposes, including compensating for sensor drift, extracting descriptive parameters from sensor array response and preparing the feature vector for further processing. A dimensionality reduction stage projects this initial feature vector onto a lower dimensional space in order to avoid problems associated with high-dimensional, sparse datasets and redundancy. The resulting low-dimensional feature vector is further used to solve a given prediction problem, generally classification, regression or clustering.

The problem of identifying an unknown sample as one from a previously learned odorant set is addressed in classification tasks. In regression tasks, the aim is to predict a set of properties (e.g., concentration for an analyte in a complex mixture or the value of a sensory panel descriptor) from other variables. Finally, in clustering tasks the goal is to learn the structural relationship among different odorants. A final step, sometimes overlooked, is the selection of models and parameter setting and the estimation of true rates for a trained model by means of validation techniques.

## 2. Material and Methods

To perform the measurements of wine samples, a home-made and home-developed electronic nose was used. The e-nose and data processing system are described in this section.

### 2.1. Wine Samples

A total of 26 wines (19 red and 7 white) from different regions were tasted by a sensory panel and analyzed by the electronic nose for the experiment. A list of the wine samples is shown in Table 1. Wine samples were acquired in specialized shops and supplied by collaborator wine cellars.

### 2.2. Sensory Panel

A group of 30 people with previous experience in wine analysis were trained in recognizing 45 aromatic compounds from wine. In this training, all compounds were presented to the panelists at their threshold concentration in water and in test wines [3]. Wines were presented in random order to the panelists. No information was given to the assessors about the origin of the samples and no colored lighting assessed panelists. The wine tasting took place in an air-conditioned room (21 °C) with isolated booths. Judges assessed the aroma using a tasting evaluation sheet that included 10 sensory descriptors (herbaceous, fruity, flower-like, spicy, vegetal, phenolic, microbiologic, chemical, oxidation and woody-like) and 5 quality parameters (wine quality, aromatic intensity, alteration, persistence and off-flavours). The different terms were evaluated in a scale from 1 to 5 (1, null, very weak; 2, weak; 3, medium; 4, strong; 5, very strong). The average of all the panelists was calculated to build the prediction models. All the sensory evaluations were realized under Spanish Standardization Rules (UNE) [4].

### 2.3. Electronic Nose

**Figure 2.**
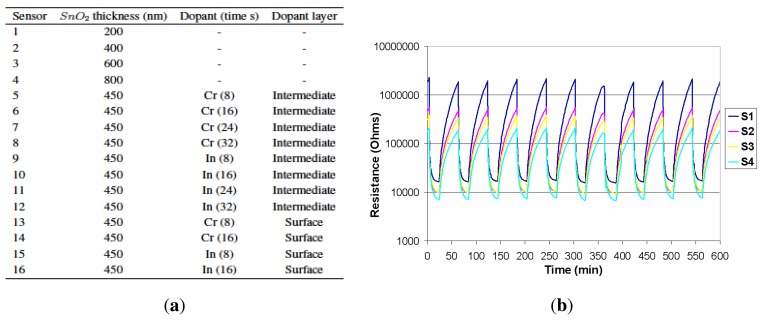
Typical transient response and doping characteristics of the gas sensors. (**a**) Sensor array composition; (**b**) Typical transient response of four sensors of the array.

**Figure 3.**
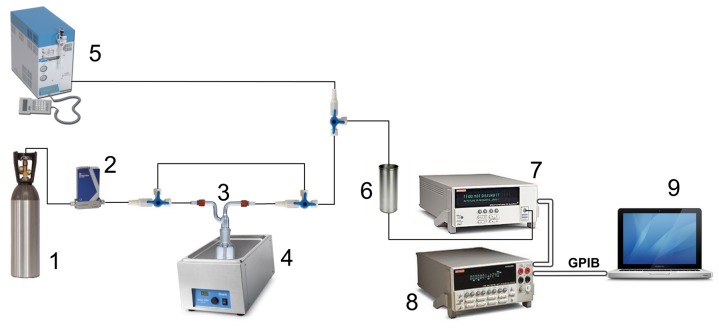
Measurement setup: 1. Nitrogen bottle; 2. Mass Flowmeter Controller; 3. Dreschel bottle with sample; 4. Thermostatic bath; 5. Tekmar 3100 purge and trap; 6. Sensors cell; 7. Digital multimeter; 8. Multiplexer; 9. Computer.

An array of 16 thin film tin oxide sensors was prepared by RF sputtering onto alumina and doped with chromium and indium. Sensor thickness varied between 200 and 800 nm. Some sensors were doped with Chromium and Indium, either on the surface or in an intermediate layer. Array composition is described in Figure 2(a). The array was placed in a 24 cm^3^ stainless steel cell with a heater and a thermocouple. The operating temperature of sensors is controlled to 250 °C with a PID temperature controller. Two sampling methods were available for analysis: purge and trap and static headspace followed by a dynamic injection. In purge and trap, a Tekmar 3100 was used [5]. In headspace, 10 mL of solution were kept in a 50 mL Dreschel bottle at 30 °C for 30 minutes followed by 20 minutes carrying the volatile compounds to the sensor cell. The carrier gas was high purity nitrogen (99.9998%) at a constant flow of 200 mL/min [6]. In Figure 3, a block diagram of the measuring system is shown.

The resistance of the sensors was measured with a Keithley 2001 8 1/2 digits digital multimeter (DMM) with a Keithley 7001 40-channels multiplexer connected to the personal computer through a GPIB interface. More details of the system can be found in [7]. Responses of the individual sensors are defined relative to the minimum resistance to a 12% (V/V) solution of ethanol in deionised water for all measurements (calibration solution). The response of the sensors is low, so no special requirements are needed in instrumentation. Two measurements are made each minute, and it is enough to obtain the response curve profile for data analysis. Figure 2(b) shows the typical transient responses of four chemoresistive sensors of the array, operating at 250 °C, exposed towards the headspace of one of the wine samples (Pioz-W1). The response of the sensors corresponds to several pulses of 20 minutes of exposition to the tested wine flavour followed by a pure nitrogen purge for 40 minutes. These responses were stored in hard disk and processed later by the data processing system for prediction and classification purposes.

### 2.4. Data Processing System

This section shows the system designed for the analysis of e-nose responses of the wine samples under test. The aim of this system is to perform the classification of the wine samples and the prediction of the sensory panel attributes from the e-nose responses. The data collected were analyzed by means of pattern recognition techniques using commercial software packages.

Matlab^®^ [8] was used for linear methods like Independent Component Analysis (ICA) [9,10] and non-linear methods based on Artificial Neural Networks (ANNs) like backpropagation and probabilistic neural networks. Partial Least Squares Regression was performed with Unscrambler^®^ [11].

#### 2.4.1. Preprocessing and Feature Extraction

The aim of feature extraction is to find a low-dimensional mapping *f : x* ∈ ℜ*^N^* → *y* ∈ ℜ*^M^*(*M* < *N*) that preserves most of the information in the original feature vector x. In our case, a previous optimization of the method of feature extraction and selection were performed [12]; the responses of the individual sensors were defined relative to the minimum resistance to 12% (V/V) of ethanol for all the measurements:

$$\text{r}=\frac{{\text{R}}_{\text{wine}}}{{\text{R}}_{\text{calibration}}}$$

where *R_wine_* was the minimum resistance of the sensor in the measurement of wine and *R_calibration_* was the minimum resistance of the sensor in a solution of 12% of ethanol. This calibration was performed once a week, to eliminate the sensors drift [2]. Data were centered and scaled for further analysis.

#### 2.4.2. Dimensionality Reduction and Classification

The traditional method for reduction in dimensionality and visualization of multivariate measurement data is Principal Component Analysis (PCA) [13]. This work introduces the higher order statistical method called Independent Component Analysis (ICA) [12] as an alternative method of data processing for an e-nose data. Moreover, ICA is an appropriate approach for signal processing as it is demonstrated in [14].

ICA is a statistical and computational technique for revealing hidden factors that underlie sets of random variables or measurements. This technique defines a generative model or the observed multivariate data, which is typically given as a large database of samples. In the model, the data variables are assumed to be linear or nonlinear mixtures of some unknown latent variables, and the mixing system is also unknown. The aim of ICA is the decomposition for multivariate signals in statistically independent component contributions with minimum loss of information [15]. ICA can be seen as an extension to principal component analysis. While PCA is an effective method for data compression, ICA is an effective method for the extraction of independent features [16,17]. It is much more powerful technique, even, capable of finding the underlying factors or sources when classic methods fail completely.

The ICA bilinear model can be written:

(1) $$\text{X}=\text{A}{\text{S}}^{\text{T}}+\text{E}$$

where **X** (*i* × *j*) is the original experimental raw data matrix and **A** (*i* × *n*) and **S***^T^* (*n* × *j*) are the so-called mixing and source matrices, respectively, in ICA notation. In addition, **E** (*i* × *j*) is the error matrix. The *i* and *j* index are respectively the numbers of rows and columns of the data matrix **X**, and *n* the number of components included in the bilinear decomposition of Equation (1). ICA algorithms try to find “unmixing” matrix **W** according to:

(2) $${\hat{\text{S}}}^{T}=\text{WX}$$

where **S**ˆ*^T^* is the estimation of the source matrix **S***^T^*. Then, when **W** is the pseudoinverse of the mixing matrix **A**, the estimated source signal will be equal to the original source signal **S***^T^* (provided that error tends to zero):

(3) $${\hat{\text{S}}}^{T}=\text{WX}=\text{WA}{\text{S}}^{T}={\text{S}}^{T}$$

The two main assumptions of ICA are:

1. The mixing vectors in **A** are linearly independent.
2. The components in **S***^T^* are mutually statistically independent.

On the other hand regression problems constitute a more challenging domain for e-nose instruments [2]. The goal of regression is to establish a predictive model from a set of independent variables (e.g., gas sensor responses) to another set of continuous dependent variables.

Partial Least Squares regression (PLS) combines the properties of multiple linear regression and principal component analysis to produce a technique that is able to accept collinear data and separate the sample noise in order to make linear combinations in the dependent concentration matrix. PLS is the “gold standard” in chemometrics due to its ability to handle collinear data and reduce the number of required calibration samples [18].

As opposed to PCR, which extracts the “latent variables” from the directions of maximum variance in the sensor matrix Y (the eigenvectors of YTY), PLS finds the directions of maximum correlation between the sensor response matrix and the calibration mixtures matrix (Y and C) in a sequential fashion. The first PLS latent variable (t = Y*ω*) is obtained by projecting Y along the eigenvector *ω* corresponding to the largest eigenvalue of *Y^T^CC^T^Y* [19]. To find the second and subsequent latent variables, the current PLS latent variable is deflated by its ordinary least squares prediction (a simple approach to regression is to assume that the dependent variables can be predicted from a linear combination of the sensor responses) and the eigen-analysis is repeated. A stopping point for the sequential expansion is determined through cross-validation. Published works on multicomponent analysis using gas sensor arrays and PLS may be found in references [20–23].

### 2.5. Artificial Neural Networks

The discrimination of the tested wines belonging to the same class has been tackled with a pattern recognizer based on ANN providing nonlinearity in the multivariate classification performance. Two types of neural networks have been tested for the classification of the wine aroma.

Also, Leave-One-Out (LOO) cross validation was applied to check the performance of the network [24]. LOO consists in training *N* distinct nets (where *N* is the number of measurements) by using *N* − *1* training vectors, while the validation of the trained network is carried out by using the remaining vector, excluded from the training set. This procedure is repeated *N* times until all vectors are validated [25].

#### 2.5.1. Backpropagation Networks

Backpropagation algorithm is the most suitable learning rule for multi-layer perceptrons. It involves two stages: firstly, a feedforward stage where the exterior input information on the input nodes is propagated forward in order to compute the output information indicators at the output unit, and secondly, a backward phase where alterations to the connection weights are adjusted based on the differences between the computed and the actual indications at the output units [26,27].

The information processing that it carry out is basically an approximation of a mapping or function:

(4) $$f:D\subset R^{n}\to R^{m}$$

by means of training over a set of example mapping, *y_k_* = *f*(*y_k_*), where *k* = 1, 2, 3…, *N*

The backpropagation network architecture used has a learning rate of 0.01 and training times less than 3 minutes. Also, it is formed by three layers: the input layer has 16 neurons corresponding to the 16 sensors, a variable number of neurons with *tansig* function for the hidden layer, and nine neurons with *hardlim* function for the output layer, the same number of existing classes.

#### 2.5.2. Probabilistic Networks

PNN is a kind of feedforward neural network. The original PNN structure is a direct neural network implementation of Parzen nonparametric Probability Density Function (PDF) estimation and Bayes classification rule [28,29]. The standard training procedure of PNN requires a single pass over all the patterns of the training set [29].

If *X* ∈ *R^d^* is a *d*-dimensional pattern vectors and its associated class is *i* ∈ (*S*_1_, *S*_2_, *S*_3_, …, *S_k_*), where *k* is the number of possible classes, and *a posteriori* probability *P_r_*(*S_i_*|*x*), that is from class *S_i_*, is by Bayes' rule:

(5) $$P_{r}(S_{i}|x)=\frac{P_{r}(x|S_{i})P_{r}(S_{i})}{P(x)}$$

where *P_r_*(*x*|*S_i_*), with *i* = 1, 2, 3, …, *k* is *a priori* pdf of the pattern in classes to be separated. And *P_r_*(*S_i_*) with *i* = 1, 2, 3, …, *k* are *a priori* probabilities of the classes. *P*(*x*) is assumed to be a constant.

The decision rule is to select class *S_i_* for with *P_r_*(*S_i_*|*x*) is maximum. This will happen if for all *j* ≠ *i*

(6) $$P(x|S_{i})\;P_{r}\;(S_{i})>P(x|S_{i}\;)P_{r}(S_{j})$$

A Probabilistic Neural Network (PNN) composed of three layers, with radial basis transfer functions in the hidden layer and a competitive one in the output [2], was used for classification purposes.

## 3. Results and Discussion

Wine samples were tasted by the sensory panel and analyzed by the electronic nose. Only headspace sampling method have been used in electronic nose because of the purge and trap did not offer good prediction ability due to the concentration and elimination of several chemical compounds in the trap [30]. Matlab® program was used for preprocessing and feature extraction. This program automatically processes the measurements files and generates the output vectors for dimensionality reduction. Besides, the program uses the neural networks toolbox and it performs the dimensionality reduction using PCA, LDA or ICA and classification with several types of Neural Networks (BackPropagation and Probabilistic Neural Networks). Finally it generates the corresponding plots and the confusion matrix obtained with the validation of ANNs. A summary of the results obtained in these analyses is shown in the following paragraphs.

### 3.1. Sensory Panel Responses

Taste of the samples was performed by the panel. The data obtained were processed and the average of all the panelists was calculated for each wine and descriptor. The sensory attributes obtained for young white, young red and oak aged wines are shown in Table 2. In young white wines the flower and fruity aromas predominate, whereas in young red wines the fruity and spicy aromas predominate, and the fruity, spicy and woody-like aromas predominate in oak aged wines.

### 3.2. E-Nose Response

As mentioned in Section 2.3, Figure 2 represents the typical transient responses of four chemoresistive sensors, operating at 250 °C, exposed towards the headspace of the blank wine. The response of the sensors corresponds to several pulses of 20 minutes of exposition to the sample of wine, followed by a pure nitrogen purge for 40 minutes. When electrovalves are switched on and wine aroma is carried to the sensors cell, the resistance of the sensors decrease and when electrovalves are switched off, the response increase to the equilibrium values.

**Figure 4.**
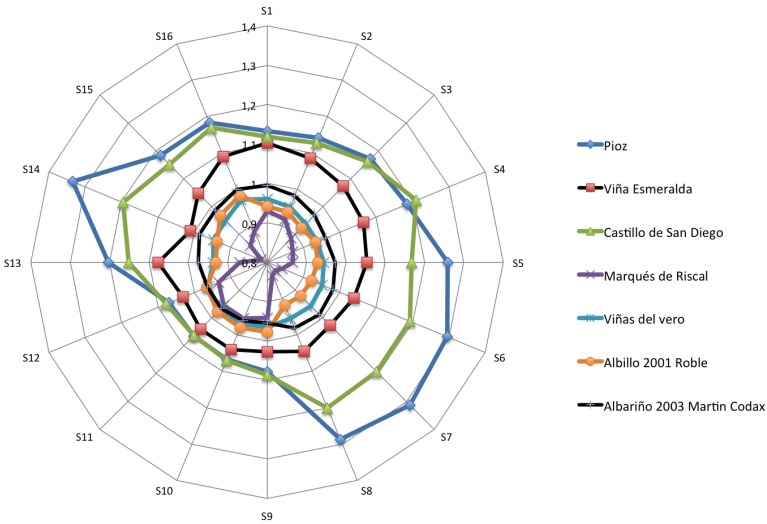
Polar plots of the variation in sensor resistance for the white wines.

**Figure 5.**
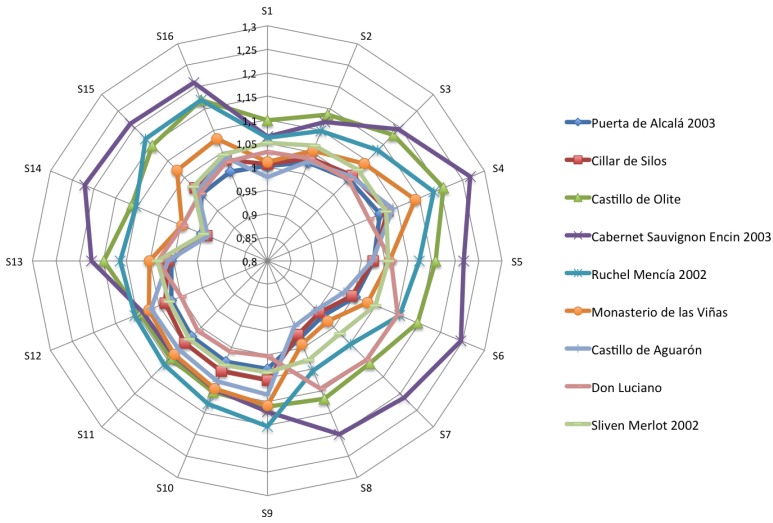
Polar plots of the variation in sensor resistance for the red wines.

**Figure 6.**
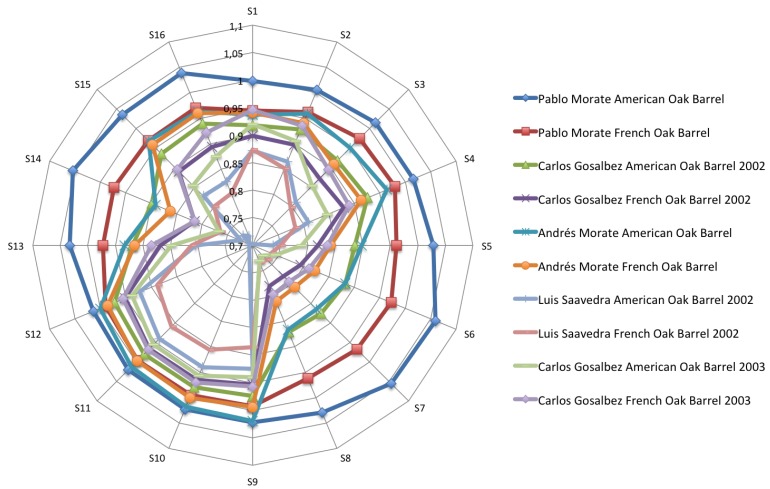
Polar pSlots of the variation in sensor resistance for the aged red wines.

Figures 4–6 show the radial plot of the 16 sensors responses to the three sets of samples measured: white, red and aged wines respectively. It can be noticed that the sensors give different signal for each different sample, illustrating the discrimination capabilities of the array.

No relation among sensors response and aromatic profile of each wine can be established according to Figures 4–6 and a classification of samples cannot be performed. The variation in response intensity is due to different headspace composition of the wines. Each sample has its characteristic organic volatile compounds profile.

### 3.3. Dimensionality Reduction and Classification

**Figure 7.**
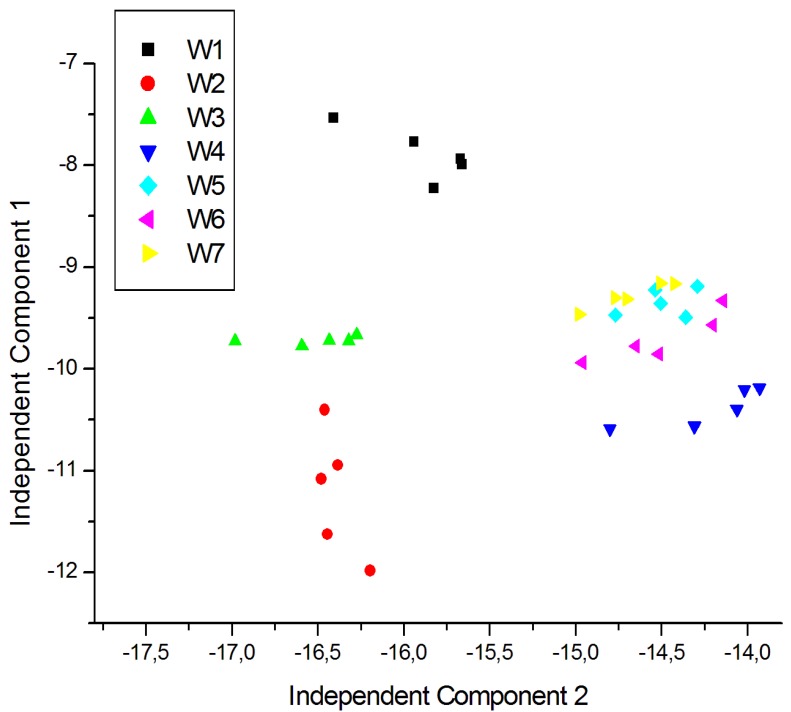
ICA plot of the young white wine measurements.

**Figure 8.**
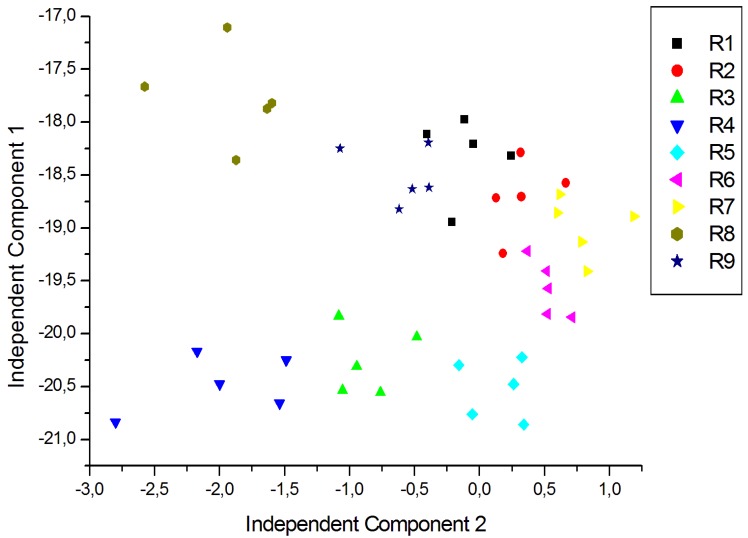
ICA plot of the young red wine measurements.

**Figure 9.**
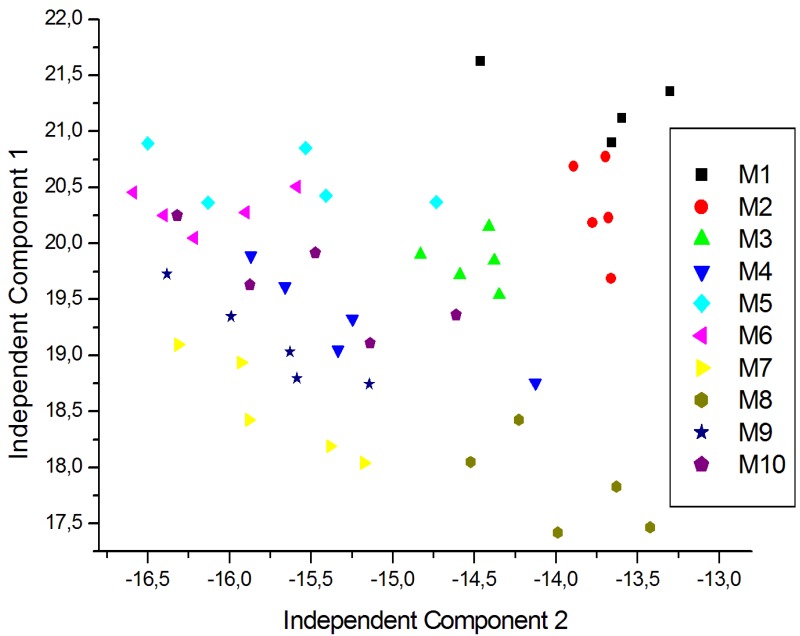
ICA plot of the aged red wine measurements.

For a better visualization of the data and to eliminate redundancy, dimensionality reduction techniques are generally used. In this case ICA was carried out using signals corresponding to five repeated exposures collected in different days. The plot of the two first Independent Components for the measurements of young white, young red and aged red wines are shown in Figures 7, 8 and 9 respectively. As shown in Figure 7, the clusters corresponding to each wine are well separated except some partial overlapping between samples of W7 and W5. The young white wines are clearly separated among them. Figure 8 shows the ICA plot for young red wines, where a partial overlapping among some samples of R1, R2 and R9 can be observed. The clusters of the other wines are well separated. Figure 9 shows the ICA plot for aged red wines. In this figure there are several overlapping zones between some wines due to the similarity of the wines. Some of these zones correspond to measurements of the same wine but aged in a different type of oak barrel, e.g., M5–M6 and M9–M10. On the contrary, other wine samples, such as M1–M2, M3–M4 and M7–M8 are well separated.

A regression model to estimate sensory panel indicators from electronic nose was built by partial least squares regression (PLS). Models were cross-validated by the leave-one-out method. Preprocessing, modelling and validation were performed with Unscrambler^®^. Parameters used to evaluate the models prediction ability were: root mean square prediction error and correlation coefficient between real and predicted Y variables. All variables were normalized prior to the analysis.

#### 3.3.1. Sensory Data *versus* E-Nose Data

The correlation coefficients and mean squared error values calculated for all the parameters in this extraction technique are listed in Table 3.

**Figure 10.**
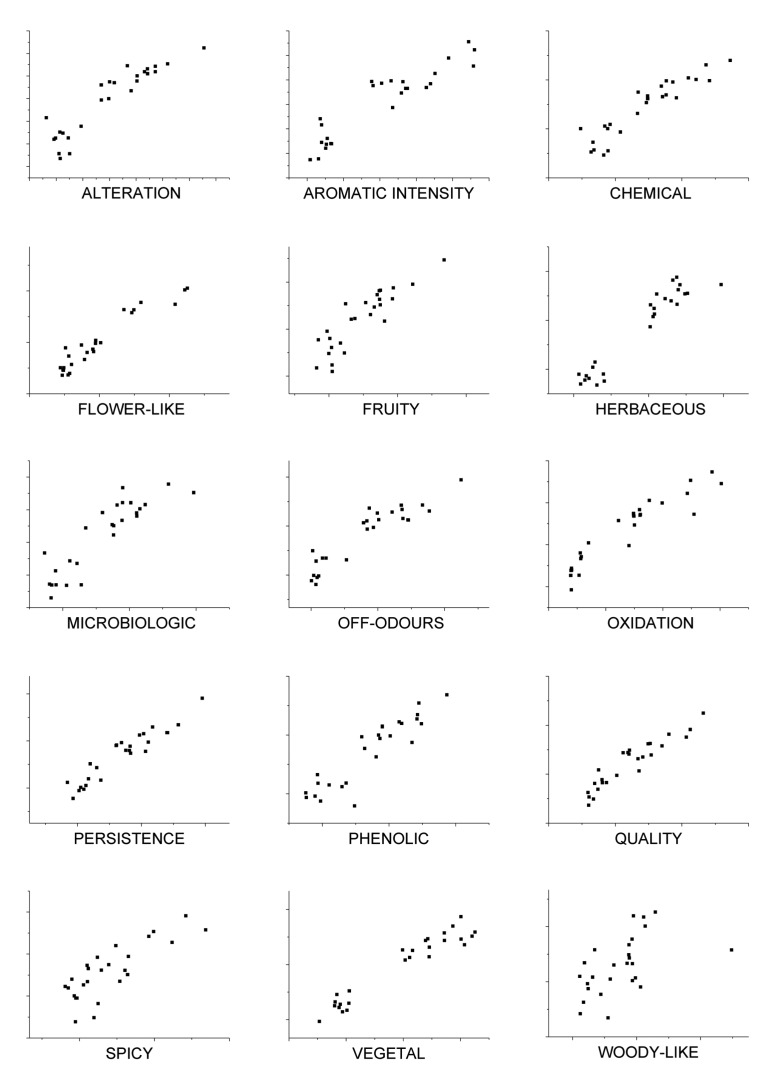
Sensory panel attributes estimated by PLS model (y) *vs.* real values (x).

These results show that good correlation coefficients and mean squared error values are obtained except for woody-like parameter. Nevertheless, results could be improved by analyzing more wine samples with sensory panel and e-nose in order to train the model and obtain more accuracy in the predictions. Models were built in order to predict the sensorial descriptors and they exhibit good prediction ability, with correlations coefficients from 0.50 to 0.95 and mean squared error values between 4.31 ×10^−4^ and 1.29 ×10^−3^. Figure 10 shows the relationship between estimated (y-axis) and real values of sensorial descriptors (x-axis) based on the PLS model calculated from e-nose responses using headspace method. Validation points are shown in figures obtained with cross-validation. As shown in Figure 10, there is a close relationship between sensory panel attributes from sensory evaluation and those estimated by the PLS model. Therefore, a good prediction of these variables could be performed from the e-nose measurements.

### 3.4. Artificial Neural Networks

Two types of neural networks were used for classification: Backpropagation and Probabilistic Neural Networks. In general the output of the neural network is given as a confusion matrix. From the confusion matrix a success rate is defined as the number of correct classified measures in each class over the total number of measures in this class.

#### 3.4.1. Backpropagation Networks

In the training of feedforward networks, several numbers of neurons in the hidden layer have been tested. The optimal number turned out to be 14 neurons. The network was trained with the data obtained with the wine samples measurements. Leave-one-out (LOO) validation was performed in order to check the performance of the network. The classification success was 97% for young white wines, 87% for young red wines and 84% for aged red wines. As an example, the confusion matrix obtained for white wines is shown in Table 4.

#### 3.4.2. Probabilistic Networks

Probabilistic neural network were trained with the same data than backpropagation networks. Leave-one-out (LOO) validation was also performed. The classification success was 94% for young white wines, 84% for young red wines and 82% for aged red wines.

## 4. Conclusions and Future Works

An electronic nose has been designed in this work for prediction and classification of wine samples. This e-nose consist of a gas sensor array combined with Partial Least Squared and a pattern recognition system based on Independent Component Analysis for dimensionality reduction. Results obtained through wine headspace technique have been used for predicting the sensory panel descriptors of the wines analyzed. In general, good results have been obtained for the correlation between sensory panel and e-nose due to the similarity between this sampling system and the natural process of taste in humans. This is a consequence to the global response of receptors (human nose and chemical sensors) to the whole aroma, allowing to predict wine quality parameters and sensory descriptors of sensory panel.

The signals recorded from the multisensor array exposed to the aroma of the wine samples have also been used for classifying these samples through ICA as explorative techniques and dimensionality reduction combined with a pattern recognizer based on ANN. Results show classification success ratios higher than 87% for all types of wine.

Finally, it is important to remark that, results could be improved by measuring more wine samples in order to increase the database and to train the networks and model for classification and prediction. Samples should be selected in order to present a wide range in the parameters to predict.

## Figures and Tables

**Table 1.** Wine samples tasted by the sensory panel and measured by the e-nose.

**Num.**	**Wine**	**Type of wine**
W1	Pioz (Guadalajara, Spain)	Young white
W2	Viña Esmeralda (Miguel Torres, Spain)	Young white
W3	Castillo de San Diego (B. Barbadillo, Spain)	Young white
W4	Marqués de Riscal (Rioja, Spain)	Young white
W5	Viñas del Vero (Somontano, Spain)	Young white
W6	Don Álvaro de Luna Albillo 2001 (Madrid, Spain)	Young white
W7	Albariño 2003 (B. Martín Códax, Spain)	Young white
R1	Puerta de Alcalá 2003 (Vinos Jeromín, Madrid, Spain)	Young red
R2	Cillar de Silos (Bodegas de la Villa, Spain)	Young red
R3	Castillo de Olite (Bodegas Artesanas, Spain)	Young red
R4	Cabernet Sauvignon Encin 2003 (Madrid, Spain)	Young red
R5	Ruchel Mencía 2002 (B. Majlu, Spain)	Young red
R6	Monasterio de las Viñas (Cariñena, Spain)	Young red
R7	Castillo de Aguarón (Carineña, Spain)	Young red
R8	Don Luciano (B. García Carrión, Spain)	Young red
R9	Sliven Merlot 2002 (Bulgary)	Young red
M1	Pablo Morate American Oak Barrel (Madrid, Spain)	Oak Barrel aged
M2	Pablo Morate French Oak Barrel (Madrid, Spain)	Oak Barrel aged
M3	Carlos Gosalbez American Oak Barrel 2002 (Madrid, Spain)	Oak Barrel aged
M4	Carlos Gosalbez French Oak Barrel 2002 (Madrid, Spain)	Oak Barrel aged
M5	Andrés Morate American Oak Barrel (Madrid, Spain)	Oak Barrel aged
M6	Andrés Morate French Oak Barrel (Madrid, Spain)	Oak Barrel aged
M7	Luis Saavedra American Oak Barrel 2002 (Madrid, Spain)	Oak Barrel aged
M8	Luis Saavedra French Oak Barrel 2002 (Madrid, Spain)	Oak Barrel aged
M9	Carlos Gosalbez American Oak Barrel 2003 (Madrid, Spain)	Oak Barrel aged
M10	Carlos Gosalbez French Oak Barrel 2003 (Madrid, Spain)	Oak Barrel aged

**Table 2.** Wine samples measured by the e-nose and tasted by the sensory panel.

	**Herbaceous**	**Flower-like**	**Fruity**	**Spicy**	**Vegetal**	**Phenolic**	**Microbiologic**	**Chemical**	**Oxidation**	**Woody-like**	**Alteration**	**Persistence**	**Average intensity**	**Off-flavours**	**Wine quality**
Pioz	1.63	2.29	2.59	1.71	1.82	1.41	1.35	1.53	1.59	1.18	1.35	2.53	2.59	1.86	2.37
Viña Esmeralda	1.69	3.47	3.59	1.41	1.41	1.18	1.29	1.24	1.12	1.06	1.12	3.53	3.76	1.2	3.71
Castillo de San Diego	1.56	2.33	2.06	1.5	1.33	1.28	1.39	1.28	1.33	1	1.5	2.33	2.59	1.48	2.44
Marqués de Riscal	2	3.28	2.17	1.61	1.89	1.28	1.17	1.44	1	1	1.44	3.06	3.85	1.59	3.44
Malvar Encín 2003	1.38	3.29	1.94	1.18	1.47	1.24	1.24	1.35	1.24	1.06	1.24	2.94	3.06	1.35	3.08
Viñas del Vero	1.61	2.5	2.33	1.78	1.67	1.5	1.78	1.39	1.17	1.78	1.61	2.72	3.37	1.8	2.8
Don Álvaro de Luna	1.41	2.11	1.67	2.17	1.39	1.56	1.44	1.5	1.56	3.11	1.5	2.83	3.52	1.57	3.13
Martín Codax	1.5	3.56	3	1.22	1.56	1.17	1.06	1.06	1.17	1.11	1.28	3.22	3.87	1.24	3.52
Puerta de Alcalá 2003	1.41	1.32	2.27	2.05	1.55	1.45	1.32	1.23	1.14	1.23	1.18	2.45	2.76	1.36	2.52
Cillar de Silos	1.41	1.5	2.64	1.77	1.82	1.27	1.41	1.23	1.09	1.41	1.32	3.09	3.35	1.67	3.06
Garnacha Encín 2003	1.55	1.45	2.09	1.55	1.41	1.32	1.18	1.5	1.41	1.18	1.55	2.5	2.64	1.68	2.45
Castillo de Olite	1.41	1.45	2.45	2.32	1.68	1.55	1.27	1.32	1.82	1.91	1.45	2.77	3.03	1.67	2.73
Cabernet Sauvignon Encín 2003	1.38	1.44	1.88	1.31	1.38	1.56	1.63	1.69	1.75	1.06	1.94	2.5	3.06	2.21	2.29
Ruchel Mencía 2002	1.63	1.19	1.81	2	1.56	1.56	1.25	1.13	1.56	1.5	1.38	2.38	2.88	1.63	2.52
Monasterio de las Viñas	1.63	1.63	2.38	1.81	1.94	1.38	1.44	1.06	1.19	1.31	1.44	2.56	3	1.38	2.67
Castillo de Aguarón	1.56	1.25	2.38	1.69	1.75	1.31	1.31	1.13	1.13	1.13	1.06	2.56	2.88	1.27	2.67
Don Luciano	1.38	1.5	2.38	1.44	1.81	1.31	1.25	1.13	1.13	1.44	1.13	2.69	3	1.25	2.85
Sliven Merlot 2002	1.69	1.44	2.31	2.5	1.56	1.75	1.25	1.25	1.25	1.75	1.06	2.81	3.29	1.31	2.88
Pablo Morate American Oak Barrel	1.5	1.19	1.94	2.63	1.38	1.34	1.38	1.25	1.19	3.5	1.19	2.81	3.55	1.23	3.23
Pablo Morate French Oak Barrel	1.31	1.25	1.88	2.5	1.38	1.44	1.31	1.13	1.19	2.81	1	2.94	3.1	1.17	3.04
Carlos Gosalbez American Oak Barrel 2002	1.25	1.31	2.38	2.25	1.56	1.75	1.69	1.44	1.06	2.88	1.5	3.25	3.44	1.75	3.06
Carlos Gosalbez French Oak Barrel 2002	1.38	1.38	2.31	2.56	1.56	1.5	1.56	1.44	1.06	2.94	1.31	3.34	3.31	1.27	3.32
Andrés Morate American Oak Barrel	1.22	1.28	2.39	2.11	1.17	1.33	1.44	1.28	1.06	2.86	1.11	3.19	3.34	1.19	3.31
Andrés Morate French Oak Barrel	1.33	1.5	2.22	2.22	1.44	1.89	1.39	1.39	1.17	3.14	1.33	3.31	3.46	1.15	3.44
Luis Saavedra American Oak Barrel 2002	1.6	1.3	2.4	2.23	1.43	1.8	1.4	1.47	1.2	2.87	1.2	3.13	3.46	1.4	3.03
Luis Saavedra French Oak Barrel 2002	1.6	1.47	2.27	2.33	1.47	1.47	1.43	1.57	1.29	3.23	1.23	3.07	3.27	1.22	3.17
Carlos Gosalbez American Oak Barrel 2003	1.47	1.53	2.8	2.4	1.4	1.6	1.73	1.27	1.07	2.97	1.2	3.6	3.56	1.24	3.56
Carlos Gosalbez French Oak Barrel 2003	1.43	1.6	2.67	2.17	1.53	1.47	1.6	1.4	1.07	2.93	1.13	3.5	3.34	1.33	3.42

**Table 3.** Correlation coefficients and mean squared error values between the sensory panel descriptors and e-nose variables in validation.

**Sensory panel**	**Correlation Coefficient**	**Mean Squared Error**
Alteration	0.919630	0.000560
Aromatic intensity	0.923276	0.001061
Chemical	0.922897	0.000431
Flower-like	0.952748	0.001094
Fruity	0.879096	0.001142
Herbaceous	0.953079	0.000479
Microbiologic	0.882264	0.000499
Off-flavours	0.915963	0.000692
Oxidation	0.932035	0.000552
Persistence	0.940271	0.000701
Phenolic	0.908509	0.000464
Quality	0.931305	0.000852
Spicy	0.799893	0.000744
Vegetal	0.959348	0.000489
Woody-like	0.508733	0.001286

**Table 4.** Confusion matrix obtained in validation of network with white young wines.

	**W1**	**W2**	**W3**	**W4**	**W5**	**W6**	**W7**
W1	5	0	0	0	0	0	0
W2	0	5	0	0	0	0	0
W3	0	0	5	0	0	0	0
W4	0	0	0	5	0	0	0
W5	0	0	0	0	4	1	0
W6	0	0	0	0	0	5	0
W7	0	0	0	0	0	0	5
